# Rubicon-deficiency sensitizes mice to mixed lineage kinase domain-like (MLKL)-mediated kidney ischemia-reperfusion injury

**DOI:** 10.1038/s41419-022-04682-3

**Published:** 2022-03-14

**Authors:** Wulf Tonnus, Sophie Locke, Claudia Meyer, Francesca Maremonti, Lena Eggert, Anne von Mässenhausen, Stefan R. Bornstein, Douglas R. Green, Andreas Linkermann

**Affiliations:** 1 Division of Nephrology, Department of Internal Medicine 3, University Hospital Carl Gustav Carus at the Technische Universität Dresden, Dresden, Germany; 2 Biotechnology Center, Technische Universität Dresden, 01307 Dresden, Germany; 3 Diabetes and Nutritional Sciences, King’s College London, London, UK; 4 Center for Regenerative Therapies, Technische Universität Dresden, Dresden, Germany; 5 Paul Langerhans Institute Dresden of Helmholtz Centre Munich at University Clinic Carl Gustav Carus of TU Dresden Faculty of Medicine, Dresden, Germany; 6 Lee Kong Chian School of Medicine, Nanyang Technological University, Singapore, Singapore; 7 Department of Immunology, St. Jude Childrens Research Hospital, Memphis, TN USA

**Keywords:** Cell death, Kidney diseases

## Abstract

The cytosolic protein rubicon (RUBCN) has been implicated in the removal of necrotic debris and autoimmunity. However, the role of RUBCN in models of acute kidney injury (AKI), a condition that typically involves necrotic kidney tubules, was not investigated. Here, we demonstrate that RUBCN-deficient mice are hypersensitive to renal damage induced by ischemia-reperfusion injury (IRI) and cisplatin-induced AKI. Combined deficiency of RUBCN and mixed lineage kinase domain-like (MLKL) partially reversed the sensitivity in the IRI model suggesting that the absence of RUBCN sensitizes to necroptosis in that model. Necroptosis is known to contribute to TNFα-induced severe inflammatory response syndrome (SIRS), but we detected no statistically significant difference in overall survival following injection of TNFα in RUBCN-deficient mice. We additionally generated RUBCN-deficient mice which lack gasdermin D (GSDMD), the terminal mediator of pyroptosis, but no reversal of the AKI phenotype was observed. Finally, and in contrast to the previous understanding of the role of RUBCN, we did not find a significant autoimmune phenotype in RUBCN-deficient mice, but detected chronic kidney injury (CKD) in aged RUBCN-deficient mice of both sexes. In summary, our data indicate that RUBCN-deficient mice are hypersensitive to kidney injury.

## Introduction

Phagocytosis of dead or dying cells was studied in detail over the last decade [1, 2]. The protein rubicon (RUBCN) was initially demonstrated as an autophagy regulator associated with the protein Beclin-1 [3, 4], and later demonstrated to be involved in endosomal maturation [5, 6]. It is also known that RUBCN recruits NADPH oxidases to participate in the defence against bacteria [7], such as *Listeria monocytogenes* [8]. Recently, RUBCN was demonstrated to be involved in a specific type of cellular uptake of extracellular content referred to as LC3-associated phagocytosis (LAP) [9–12]. This process is thought to be critically important in the removal of necrotic debris and may be critical for tissues to allow regeneration following acute injury while avoiding autoimmunity [13].

Acute kidney injury (AKI) is associated with the loss of nephrons and therefore may fundamentally contribute to the progression of chronic kidney disease [14–16]. As a clinical consequence of progressive nephron loss, end-stage renal disease (ESRD) requires dialysis treatment or renal transplantation. Acute tubular necrosis is a hallmark condition of AKI, and we reasoned that failure to remove necrotic debris following AKI might affect renal injury in murine models of AKI. We and others recently demonstrated that mixed lineage kinase domain-like (MLKL)-mediated necroptosis is involved in acute kidney injury in mouse models [17–20] and in humans [21]. Likewise, other forms of regulated necrosis, such as ferroptosis [22, 23] and gasdermin D (GSDMD)-mediated pyroptosis [24], are potentially involved in AKI.

Here, we demonstrate that RUBCN-deficient mice are hypersensitive to AKI, an effect that is partially reversed on the combined loss of RUBCN and MLKL, but not RUBCN and GSDMD. In addition, we find aged RUBCN-deficient male mice to develop chronic kidney disease and proteinuria.

## Material and methods

### Reagents

**Table:** 

REAGENT or RESOURCE	SOURCE	IDENTIFIER
*Antibodies*		
Rubicon (D9F7) rabbit monoclonal antibody	Cell Signalling	8465
β-actin antibody	Cell Signaling	3700 S
Anti-mouse IgG; HRP-linked antibody	Cell Signaling	7076 S
Anti-rabbit IgG; HRP-linked antibody	Cell Signaling	7074 S
*Compounds, chemicals, and kits*		
zVAD fmk	BD Biosciences	550377
TNFα	Biolegend	570108
Nec-1s	Merck Millipore	5. 04297. 0001
7-AAD	BD Biosciences	559925
Annexin-V-FITC	BD Biosciences	556420
Annexin-V-binding buffer	BD Biosciences	556454
LDH-release assay	Promega	G1780
Bradford assay	Thermo Fisher Scientific	23225
ECL^™^ Prime Western Blotting System	Thermo Fisher Scientific	GERPN2232
Opti-MEM® I Medium	Thermo Fisher Scientific	31985062
Lipofectamine™ RNAiMAX	Thermo Fisher Scientific	13778075
Silencer select pre-designed siRNA, RIKEN cDNA 1700021K19 gene (Rubicon)	Thermo Fisher Scientific	s104761
Silencer® Select Negative Control	Thermo Fisher Scientific	4390843
Nonident P-40 (NP-40) substitute	Roche Diagnostics GmbH	11754599001
Ethylendiamintetraessigsäure (EDTA)	Carl Roth GmbH & Co. KG	8040.3
Sodium dodecyl sulfate (SDS)	Clinical Pharmacy of the University Hospital	N/A
Tris(hydroxymethyl)aminomethane (Tris base)	Carl Roth GmbH & Co. KG	4855.1
phenylmethyl sulfonyl fluoride (PMSF)	Roche Diagnostics GmbH	11359061001
cOmplete Mini Protease inhibitor cocktail tablets	Roche Diagnostics GmbH	11836153001
PhosSTOP Phosphatase inhibitor cocktail tablets	Roche Diagnostics GmbH	4906845001
ROTILoad Protein loading buffer, reducing	Carl Roth GmbH & Co. KG	K929.1
Precision plus protein dual color standards	Bio-Rad Laboratories Inc.	161–0374
Glycine	Sigma–Aldrich Chemie GmbH	G8898-1KG
Tween 20 (Polysorbate)	VWR International GmbH	8. 17072.1000
Methanol (99,9 %)	AppliChem GmbH	1. 06009.1011
Trans-Blot Turbo (5 x) Transfer buffer	Bio-Rad Laboratories Inc.	1704272
Ethanol denatured (>99,8%)	Carl Roth GmbH & Co. KG	K928.5
Ponceau S solution	Sigma–Aldrich Chemie GmbH	P7170
Albumin bovine Fraction V	SERVA Electrophoresis GmbH	11930.04
Nonfat dried milk powder	AppliChem GmbH	A0830, 0500
Re-Blot Plus Strong Solution (10×)	EMD Millipore Corp.	2504
Sodium chloride solution 0.9% BC	Berlin-Chemie AG	3820076
Paraformaldehyde solution 4% pH 7.4	Clinic Pharmacy of the University Hospital Carl Gustav Carus	N/A
Isofluran Baxter, liquid to produce steam for inhalation	Baxter S.A.	HDG9623
Gibco Dulbecco’s Modified Eagle Medium F-12 Nutrient Mixure without phenol red (DMEM F-12)	Thermo Fisher Scientific	11320-074
Mouse Anti-Nuclear Antibodies (ANA) Total Ig ELISA	Alpha Diagnostics International	5210
Mouse Anti-dsDNA IgA ELISA	Alpha Diagnostics International	5120-A
Lipofectamine RNAiMAX	Thermo Fisher Scientific	13778-075, 13778-150
Pierce BCA Protein Assay Kit	Thermo Fisher Scientific	23225
ECL Prime Western Blotting Detection Reagent Kit	Global Life Sciences Solutions Operations UK Ltd.	RPN2232

### Mice

Mice of the C57BL/6 N strain, further referred to as wild-type mice, were purchased from the company Charles River, Germany. *Rubicon*^*KO*^ (*Rubcn*^*KO*^) mice on a C57Bl/6 background were described previously [10] and were provided by Douglas R. Green. *Mlkl*^*KO*^ breeder pairs were described previously [25] and were kindly provided by James M. Murphy and Warren S. Alexander (The Walter and Eliza Hall Institute of Medical Research, Australia). Breeder pairs of *Gsdmd*^*KO*^ mice were provided by Feng Shao (National Institute of Biological Sciences, Beijing, China) and were described previously [26]. Double-knockout animals were generated in the animal facility in Dresden by crossing rubicon-deficient mice to MLKL-deficient or GSDMD-deficient mice. The resulting offspring was then paired up and the F2 generation genotyped. Mice double-deficient for both rubion/MLKLor rubicon/GSDMD, respectively, were then set up as homozygotic breeder pairs.

All mice have been housed in individually ventilated cages (IVC, Techniplast Deutschland GmbH) under specified pathogen-free conditions (SPF). The mice were weighed and blood samples were taken until the age of 50 weeks. After 1 year the mice were sacrificed for dissection and the organs were removed for subsequent histological analyses.

All work with animals was carried out in accordance with German animal welfare legislation and was notified and approved by the responsible authority, the Landesdirektion Dresden (TVV 57/2017) if not otherwise indicated. As required by German law, all group sizes were pre-calculated with the help of an independent statistician. The calculation basis aimed for detecting a significant difference with an alpha error of 5% and a beta error of 80%.

### Long-term assessment of rubicon-deficient mice

Blood collections were done via a puncture of the *vena facialis* or one of its branches. The samples were kept on ice for 20–30 mins and then centrifuged at 2500 × *g* for 10 min at 4 °C. Fifty microliters were diluted in 100 µl 0.9% sodium chloride solution for assessment of serum creatinine and urea levels (determined by the Clinical Chemistry and Laboratory Medicine, Universitätsklinikum Carl Gustav Carus, Technische Universität Dresden) or stored for subsequent ELISA assays.

In week 50, the mice were sacrificed and a blood glucose measurement with a blood glucose monitor was performed. Furthermore, for differential blood counts 200 µl of blood were filled into an EDTA-coated tube and sent to the Clinical Chemistry. Finally, organs were collected (kidneys, spleen, pancreas, caecum, liver, heart, lung), fixed in 4% neutral-buffered paraformaldehyde solution and transferred to 70% ethanol 24 h later. The left kidney was halved, one half was shock-frozen in a 0.5 ml tube in liquid nitrogen for western blot analysis, the other half was transferred to freezer cutting medium (tissue TEK) and shock-frozen in liquid nitrogen.

### dsDNA-Ig- and ANA-Enzyme-linked Immunosorbent Assay (ELISA)

Both enzyme-linked immunosorbent assays (ELISAs) were performed according to the manufacturer’s protocol (Alpha Diagnostics International). The samples were incubated in multiwell-plates coated with dsDNA or ANAs and visualized using an antibody coupled with horseradish peroxidase-conjugated (HRP). The hydrogen peroxide 3,3,5,5-tetramethylbenzidine (TMB) substrate was added and the optic density (OD) at 450 nm was quantified with a microwell reader (Tecan Group Ltd.).

### Histological processing

#### Periodic acid—Schiff reaction (PAS) and tubular damage score evaluation

Histological processing was carried out at the Histology Facility of BIOTEC, Technische Universität Dresden. The PAS staining was carried out using a PAS-staining Kit (MORPHISTO GmbH). For this, the tissue sections were first placed in xylene for 10 min. Subsequently, the sections were placed in fresh xylene for another 10 min. Now the samples were rehydrated in a descending alcohol series (2 × 100%,2 × 96%,70%,40%). The samples were incubated for 1 min each. The sections were then rinsed with distilled water for 1 min. Subsequently, the sections were incubated for 3 min in 1% periodic acid and washed with tap water for 30 sec afterwards. Schiff’s reagent was applied for 2.5 min. Now, the samples were transferred to Haematoxylin (sour according to Mayer) for 3 min. Afterwards washed with tap water (3 min) and distilled water for 10 sec. Then they were dehydrated in an ascending ethanol series for 1 min each (70%,3 × 96%, 2 × 100%). Now, they were placed into xylene two times for 2 min and covered in Cytoseal (Thermo Fisher Scientific Inc.).

The imaging and evaluation of the samples were carried out using the inverse microscope Axiovert 200 M (Carl Zeiss Microscopy GmbH) with the Zeiss AxioCam MRc, Color CCD camera (Carl Zeiss Microscopy GmbH) using the ZEN blue software (Carl Zeiss Microscopy GmbH). Histopathological evaluation was performed in a strictly double-blinded manner.

Tubular damage severity was assessed based on typical morphological criteria of acute renal injury. The criteria were loss of brush border, extravasation of erythrocytes, tubule dilatation, tubule degeneration, tubule necrosis and tubular cast formation. Severity was assessed on a scale of 1–10. This was determined on at least four animals per group.

**Table:** 

Score	Severity level
0	Normal, healthy tissue
1–4	Mild damage
5–6	Moderate damage
7–8	Severe damage
9–10	Very severe damage

### Bilateral kidney ischemia and reperfusion injury (IRI)

Bilateral kidney ischemia and reperfusion injury (IRI) was performed as described in detail earlier [23] and a detailed protocol is available [27]. Briefly, 8–12-weeks-old male mice were rigorously matched weight. In the experiment, they were handed over by a second person for blinding of the surgeon. After induction of narcosis, they were placed in a supine position and fixed. The abdomen was opened, the caecum and gut were carefully placed to the side. With the use of a surgical microscope (Carl Zeiss, Jena, Germany), sharp forceps were used to pinch retroperitoneal holes directly cranially and caudally in the renal pedicle. Via this access, a 100 g pressure micro serrefine (FST 18055-03) was placed on the pedicle to induce ischemia and a timer was started. The same procedure was repeated on the other side with a maximal tolerance of 60 s between both clampings. After exactly 30 min, the vascular clamps were removed. Reperfusion was determined visually for both sides before the gut was returned into the abdominal cavity. The parietal peritoneum and the cutis, respectively, were closed separately. Isoflurane application was stopped immediately thereafter, and 1 mL of prewarmed PBS was administered intraperitoneally to compensate for any possible dehydration during surgery. 0.1 µg/g buprenorphine-HCl was administered every 8 h for analgesia. After a 48 h observation period, blood was collected by retro-orbital puncture and the mice were sacrificed.

### Cisplatin-induced acute kidney injury

The model of acute kidney injury by cisplatin injection was described previously [23]. Briefly, 8–12-weeks-old female mice were rigorously matched for weight, and then received a single dose of 20 mg/kg cisplatin in 0.9% NaCl in a total volume of 400 µl intraperitoneally. The mice were then returned to their cages and monitored frequently. For analysis of kidney injury, mice were sacrificed after 48 h. Blood was taken and the kidneys were collected for further analysis. For survival assessment following cisplatin application, animals were under permanent observation and survival, as defined by the local authorities, was checked every 2–4 h. In detail, we checked for a combined endpoint of either loss of 20% body weight or the inability to maintain a body temperature higher than 28 °C or severe movement imbalances or automutilary behavior.

### TNFα-induced systemic inflammatory response syndrome

The model of TNFα-induced shock has been described in detail previously [28]. In our experiments, female 8–12-week-old mice were rigorously weight-matched before mice receiving injections of either vehicle or 25 ng murine TNFα in 200 µL PBS via the tail vein. Under blinding to the genotype, animals were under permanent observation and survival, as defined by the local authorities, was checked every 2–4 h. In detail, we checked for a combined endpoint of either loss of 20% body weight or the inability to maintain a body temperature higher than 28 °C or severe movement imbalances or automutilary behaviour.

### Analysis of serum creatinine and urea

Blood collections were done via a retro-orbital puncture. The samples were rapidly transferred to the Institute for clinical chemistry and laboratory medicine (Universitätsklinikum Carl Gustav Carus, Technische Universität Dresden) for standardized and completely blinded analysis of serum creatinine and urea levels.

### Isolation of murine kidney tubules

Isolation of tubules was performed according to a recently published protocol [23]. The kidneys of 8-week-old male mice were collected and transferred to a petri dish with 2 ml PBS. In the dish the kidneys were decapsulated. The kidney was sliced and those were transferred into a tube containing 1 ml of collagenase solution and incubated for 5 min at 37 °C and 850 rpm. This was repeated two more times and those supernatants were transferred into a new tube and the tubules were washed twice with ice-cold incubation solution. The sorting solution was added, and the tubules were distributed on a 24-well plate containing 1 ml of DMEM/F12, Thermo Fischer), supplemented with 0.01 mg/ml recombinant human insulin, 5.5 μg/ml human transferrin, 0.005 μg/ml sodium selenite (Na_2_SeO_3_) (ITS without linoleic acid, Sigma–Aldrich), 50 nM hydrocortisone, 100 U/ml penicillin, and 100 μg/ml streptomycin (Pen/Strep, Thermo Fisher). Per timepoint (0 h, 0.5 h, 1 h, 2 h, 4 h) and per mouse one well was plated. After the respective timepoints 200 µl of cell suspension was collected and stored on ice for LDH-release assay.

### Lactate dehydrogenase (LDH)-release assay

The assay was performed using the CytoTox96 Assay Kit (Promega Corporation) according to the manufacturer’s instructions. For each sample 200 µl of supernatant was taken, kept on ice and then 50 µl of lysis solution (0,8% Triton X-100) was added to the well and incubated for 45 min at 37 °C to determine the maximum LDH release. Of this, 50 µl of the supernatant was plated in triplicates on a 96-well plate. Then, the CytoTox 96® Reagent was added and incubated at room temperature for 30 min in the dark. The stop solution was added and the optical density at 490 nm was immediately determined with a plate reader.

### Cell lines and cell culture

Murine NIH-3T3 cells were purchased from the American Type Culture Collection. NIH-3T3 cells were cultured in Dulbecco’s modified Eagle medium (DMEM, Thermo Fisher) supplemented with 10% (v/v) FBS (Thermo Fisher), 100 U/ml penicillin, and 100 μg/ml streptomycin (Pen/Strep, Thermo Fisher). The cells were cultured in a humidified 5% CO_2_ atmosphere.

### siRNA-mediated knockdown of RUBCN

NIH-3T3 cells were plated in a petri dish in 15 ml antibiotic-free medium. After 24 h 180 pmol RNAi and 24 μl Lipofectamine^TM^ (Thermo Fisher) were each mixed in 1.5 ml Opti-MEM® I Medium (Thermo Fisher) without serum, combined and incubated at room temperature for 20 min before dropping the mixture on the cells. The following day, cells were harvested, plated into six-well plates and cell death assays were performed as described below. Knockdown efficacy was determined on protein level using Western Blot.

### Western blotting

Cells were lysed in ice-cold 50 mM Tris-HCl, pH 7.5, 150 mM NaCl, 1% NP-40, 5 mM EDTA supplemented with PhosSTOP^TM^ (Merck), cOmplete^TM^ (Merck), and 1 mM phenylmethyl sulfonyl fluoride (PMSF) for 30 min on ice. Insoluble material was removed by centrifugation (14,000 × *g*, 30 min, 4 °C). Protein concentration was determined using a commercial BCA assay kit according to the manufacturer’s instructions (Thermo Fisher). Equal amounts of protein (25 μg per lane) were resolved on a 4–15% gradient SDS/PAGE gel and transferred to a PVDF membrane (BIO-RAD). After blocking for 1 h at RT, primary antibody incubation (dilution of 1: 1.000 if not otherwise indicated) was performed at 4 °C overnight. Secondary antibodies (anti-mouse, HRP-linked antibody, anti-rabbit, HRP-linked antibody, Cell Signaling) were applied at concentrations of 1: 5000. Proteins were then visualized by enhanced chemiluminescence (ECL; Amersham Biosciences).

### Cell death assays

To induce necroptosis in the NIH-3T3 cell line after siRNA-mediated knockdown, cells were seeded in six-well plates and were stimulated with 20 ng/ml human TNFα (Biolegend), and 20 μM zVAD-fmk (Selleckchem). Ten micromolar Necrostatin-1s (Nec-1s, Merck KGaA) served as protection control. After the indicated timepoints, cells were collected and prepared for flow cytometry.

### Flow cytometry

Cells were harvested and the pellets were washed twice in PBS and stained with 5 μl of 7-AAD (BD Biosciences) and 5 μl of annexin-V-FITC (BD Biosciences) added to 100 μl annexin-V binding buffer (BD Biosciences). After 15 min, cells were recorded on the LSRII with the FACS Diva 6.1.1 software (BD Biosciences) and subsequently analyzed with the FlowJo v10 software (Tree Star). The flow cytometry procedure was supported by the Flow Cytometry Core Facility of the CMCB Technology Platform at TU Dresden.

### Statistical analysis

Statistical analyses were performed with Prism 8 (GraphPad software, San Diego, CA, USA). Two cohort comparisons of discrete values were analyzed by a one-sided Student’s *t*-test. If more than two groups were involved, a one-way ANOVA was performed with correction by Tukey post-hoc test. In both such datasets, the bars represent the mean ± standart deviation if not otherwise indicated. For analyses of survival data, a log-rank test was performed. Data were considered significant when *p ≤ 0.05, **p ≤ 0.01, ***p ≤ 0.001, or ****p ≤ 0.0001.

## Results

### RUBCN-deficient mice are hypersensitive to kidney ischemia-reperfusion injury

**Fig. 1:**
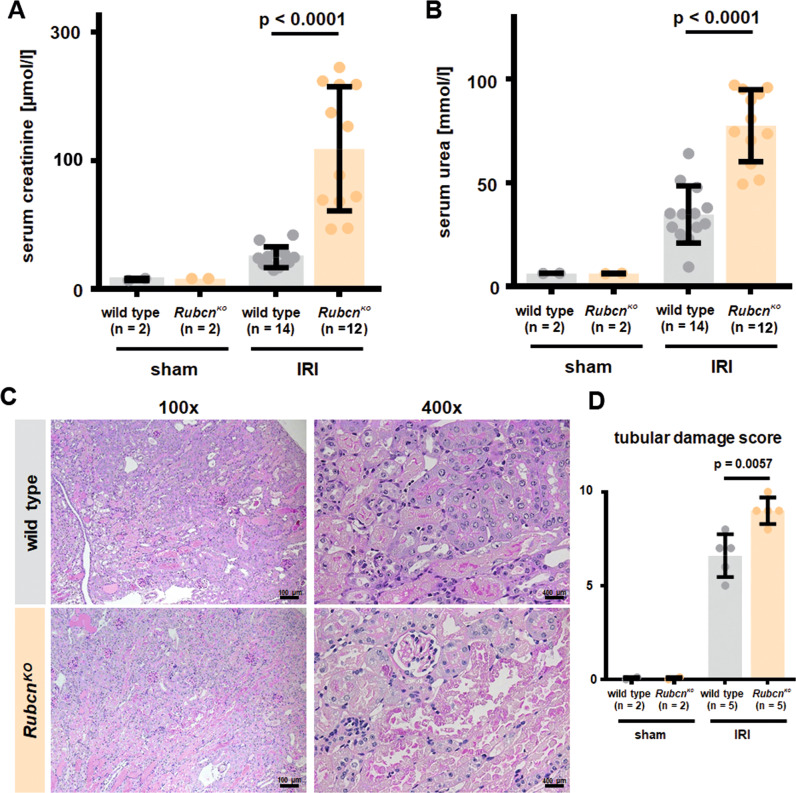
RUBCN-deficient mice are hypersensitive to kidney ischemia-reperfusion injury (IRI). Indicated numbers of 8–12-weeks-old male mice were either subjected to sham surgery or underwent bilateral kidney IRI. 48 h following the onset of reperfusion, serum creatinine (**A**) and serum urea (**B**) concentrations were measured and mice were sacrificed. Kidneys were stained for periodic acid Schiff (PAS) and two representative magnifications are demonstrated in **C** and quantified according to an established tubular damage score in **D**. Statistical analysis was performed using Student’s *t*-test (*p*-values are indicated for each experiment).

It was previously suggested that RUBCN-deficient mice might exhibit glomerular injury after the age of 20 weeks [29]. However, because necrotic debris occurs within hours in acute kidney injury (AKI)-models [30], we hypothetized that RUBCN might also be involved in the control therein. As demonstrated in Fig. 1A, RUBCN-deficient mice showed higher serum levels of the AKI marker creatinine. Along similar lines, we observed higher levels of serum urea (Fig. 1B), indicating AKI-induced uremia. Histologically, kidney cortical sections were stained for periodic acid Schiff (PAS). More tubular necrosis (Fig. 1C) and a higher tubular damage score (Fig. 1D) were detected. We previously developed a model for the assessment of tubular necrosis in freshly isolated kidney tubules [23]. In that assay, spontaneous LDH release remained unchanged in tubules isolated from RUBCN-deficient mice (Fig. S1). Another preclinical model of renal damage is cisplatin-induced AKI (CP-AKI) in which we typically investigated two timepoints in independent experiments of 48 h and 72 h following cisplatin injection. We detected a trend toward higher levels of serum creatinine (Fig. S2A) and serum urea (Fig. S2B) in the RUBCN-deficient mice, but due to high standard deviations, no statistical significance was reached despite 13 vs. 10 mice per group. However, in an entirely separate approach, we investigated the overall survival of 13 wild-type and 24 *Rubcn*^*KO*^ mice following cisplatin injection and detected a significantly shorter time to death in the RUBCN-deficient mice (Fig. S2C, *p* = 0.006). In conclusion, these data indicate that RUBCN-deficient mice are sensitive to at least two different mouse models of acute kidney injury, and that this effect cannot be directly attributed to kidney tubules.

### Combined deficiency of RUBCN and MLKL partially overcomes the sensitization of RUBCN-deficient mice in kidney IRI

**Fig. 2:**
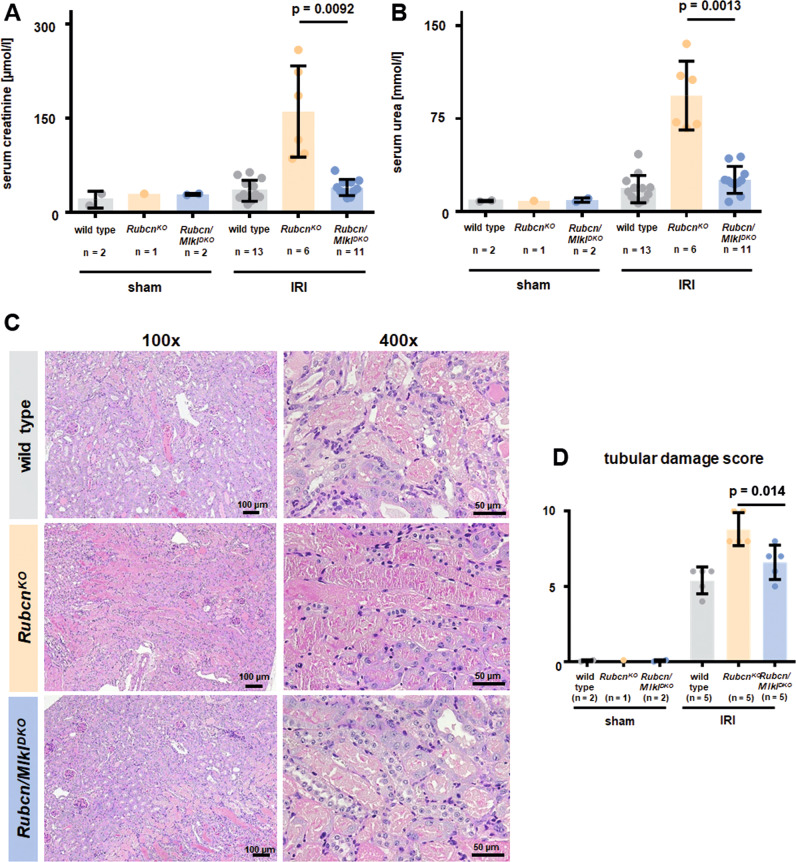
The hypersensitization of RUBCN-deficient mice is partially reversed on a combined RUBCN/MLKL-double-deficient background. Indicated 8–12-weeks-old male mice underwent bilateral kidney IRI as demonstrated in Fig. 1. 48 h following the onset of reperfusion, serum creatinine (**A**) and serum urea (**B**) concentrations were measured and mice were sacrificed. Kidneys were PAS-stained and two representative magnifications are demonstrated in **C** and quantified using the tubular damage score **D**. Statistical analysis was performed using one-way ANOVA analysis (*p*-values are indicated for each experiment).

Several signalling pathways of regulated necrosis have been implicated in the pathogenesis of AKI [22]. However, we considered it unlikely that ferroptosis is the driver of the sensitization of *Rubcn*^*KO*^ mice in AKI because renal tubules, in which ferroptosis predominates [23], were not hypersensitive. We therefore decided to investigate MLKL-mediated necroptosis and GSDMD-mediated pyroptosis as hypothetical candidate cell death pathways involved. MLKL-deficient mice, in our hands, were protected from kidney IRI [31]. Indeed, combined deficiency of RUBCN and MLKL partially reversed the significant increase in serum levels of creatinine (Fig. 2A) and urea (Fig. 2B). PAS staining of the kidney cortex is demonstrated in Fig. 2C and blinded assessment revealed less tubular damage in the *Rubcn/Mlkl*^*DKO*^ mice compared to *Rubcn*^*KO*^ (Fig. 2D). This result suggested a role for necroptosis in the sensitization of RUBCN-deficient mice to AKI.

To assess a potential role of RUBCN in classical necroptosis in general, we pre-treated NIH-3T3 cells with siRNAs against *Rubcn* for 48 h (Fig. S3A) and stimulated them for an additional 8 h with TNFα and the pan-caspase inhibitor zVAD-fmk to induce necroptosis. As a protection control, we added the specific RIPK1 inhibitor necrostatin-1s (Nec-1s) [32]. As demonstrated in Fig. S3B, RUBCN knockdown did not affect necroptosis in cell culture. To test the potential role of RUBCN in regulating necroptosis in vivo, we intravenously injected female wild-type and *Rubcn*^*KO*^ mice with recombinant TNFα to induce the well-established necroptosis model of severe inflammatory response syndrome (SIRS) [33]. As demonstrated in Fig. S4, no statistically significant difference in overall survival was detected in the RUBCN-deficient mice. In conclusion, these data strongly suggest that RUBCN does not directly regulate necroptosis, but that a non-cell-autonomous effect mediated by necroptosis might explain the partial reversal of the AKI-sensitivity of the RUBCN-deficient mice.

To next test a potential role of pyroptosis, we crossed *Rubcn*^*KO*^ mice to *Gsdmd*^*KO*^ mice (see methods for details). Combined deficiency of RUBCN and GSDMD does not affect the sensitization of RUBCN-deficient mice in kidney IRI as assessed by serum concentrations of creatinine (Fig. S5A) or urea (Fig. S5B) and did not change the morphological appearance of tubular necrosis in histology (Fig. S5C, D).

### Male RUBCN-deficient mice develop proteinuria and chronic kidney disease at 50 weeks of age while female RUBCN-deficient mice exhibit splenic atrophy

**Fig. 3:**
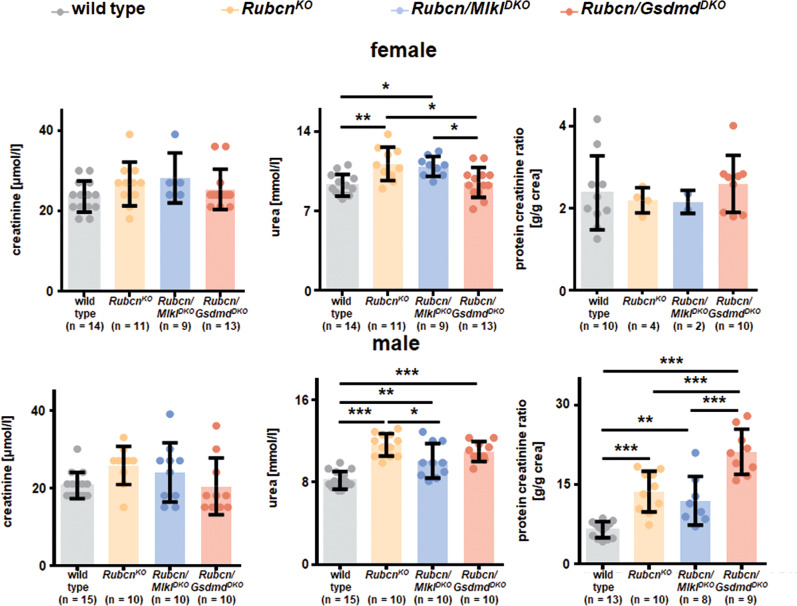
Male RUBCN-deficient mice develop slight proteinuria and mild chronic kidney disease at 50 weeks of age. Serum levels of creatinine and urea, and the urine protein to creatinine ratio are depicted for female (upper panels) and male mice (lower panels) of indicated genotypes. Note that the axis for the urine protein to creatinine ratio is higher in the male mice which develop significant proteinuria in contrast to the female littermates. Statistical analysis was performed using one-way ANOVA analysis (*p < 0.05, **p < 0.01, ***p < 0.001).

**Fig. 4:**
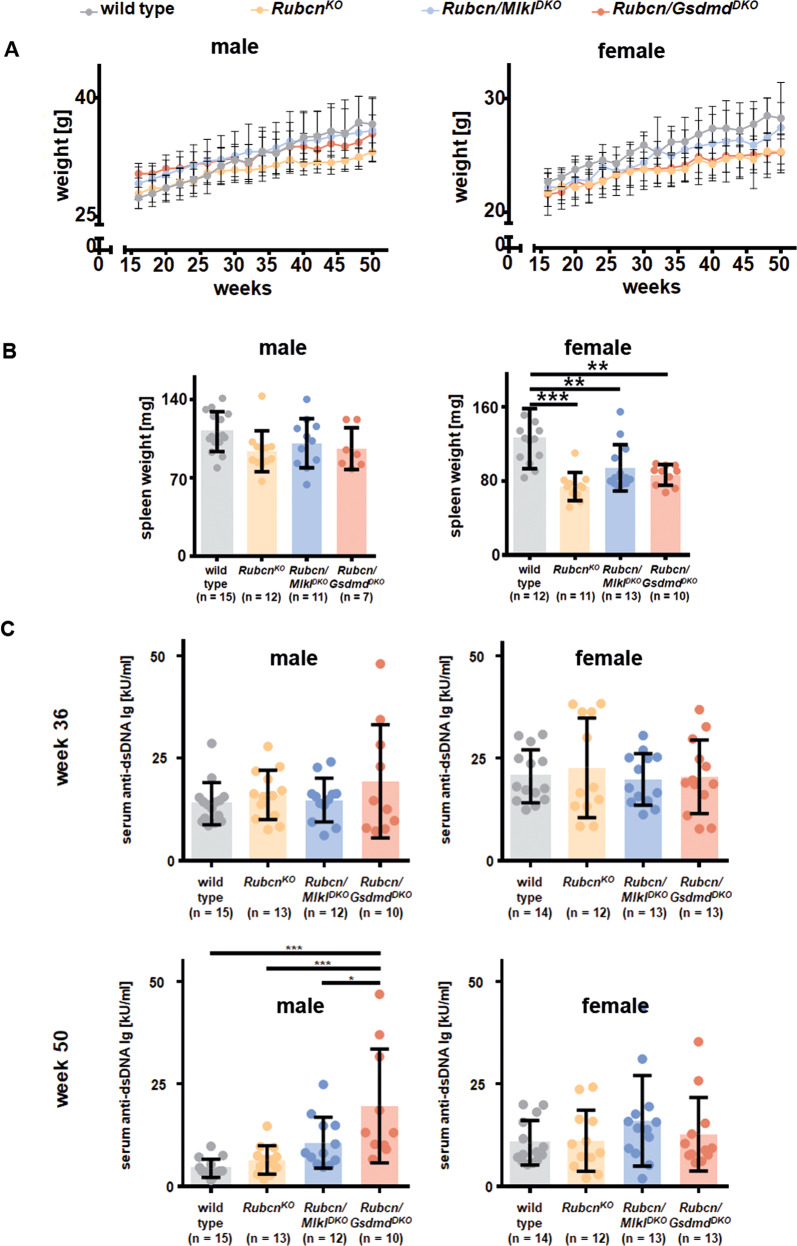
RUBCN-deficient female mice display splenic atrophy but no signs of autoimmunity at 50 weeks of age. **A** Male and female RUBCN-deficient mice fail to gain weight over their first 50 weeks of life compared to wild-type littermates (also see Fig. S6 for details). **B** In contrast to male mice, female mice show splenic atrophy at an age of 50 weeks as estimated by splenic weight. **C** Male and female wild-type mice and RUBCN-deficient littermates produce indistinguishable levels of autoantibodies against double-stranded DNA at 36 weeks and 50 weeks of age. Statistical analysis was performed one-way ANOVA analysis (*p < 0.05, **p < 0.01, ***p < 0.001).

The detection of *Rubcn*^*KO*^ mice being sensitive to AKI raised the question of the spontaneous development of chronic kidney injury in these strains. To this end, we set up a one year follow up an experiment with at least 10 mice per group of wild-type mice, RUBCN-deficient mice, *Rubcn/Mlkl*^*DKO*^ mice, and *Rubcn-Gsdmd*^*DKO*^ mice. This approach was independently followed up for both sexes. At 50 weeks of age, mice were sacrificed and serum creatinine, serum urea and urine protein-to-creatinine ratios were determined as demonstrated in Fig. 3. While both male and female RUBCN-deficient mice developed signs of chronic kidney injury (significant increases in serum urea levels), only male mice on a RUBCN-deficient background additionally presented significant proteinuria. This observation correlated with the failure to gain normal body weight in the *Rubcn*^*KO*^ and the *Rubcn-Gsdmd*^*DKO*^ mice (Fig. S6A). Interestingly, and somewhat phenocopying the partial reversal of the IRI phenotype, the failure to thrive of *Rubcn/Mlkl*^*DKO*^ mice did not reach statistical significance (Fig. S6A). Chronic kidney disease on RUBCN-deficient background did not correlate with renal anemia or erythrocytopenia in the assessment of a standard blood count (Fig. S6B). In this blood test, we additionally observed an increased number of lymphocytes, exclusively in the female mice (Fig. S6B), while serum glucose levels remained unchanged (Fig. S6C). The female lymphocytosis, unexpectedly, correlated with splenic atrophy in all RUBCN-deficient conditions (Fig. 4B), while spleen weight was indistinguishable upon all male groups investigated. Finally, and unlike previously demonstrated, we did not detect a statistically significant increase in double-stranded DNA autoantibodies in the RUBCN-deficient male or female mice at 36 or 50 weeks of age (Fig. 4C). These data indicate that the sole explanation of autoimmunity as a conclusive cause of the failure to gain weight in the *Rubcn*^*KO*^ mice is not correct. In summary, we identify RUBCN-deficient mice as a potential model for the investigation of chronic kidney injury.

## Discussion

We provide evidence for RUBCN-deficient mice to be sensitive to kidney ischemia-reperfusion injury (IRI). We further demonstrate that pyroptosis-deficiency does not reverse this sensitization, while MLKL-deficiency does. As the knockdown of *Rubcn* does not affect a standard necroptosis assay, we conclude a non-cell-autonomous involvement of MLKL. Indeed, it was recently demonstrated that metabolites released from dying cells function as tissue messengers [34], and similar mechanisms are potentially involved in models of AKI. However, we previously demonstrated that MLKL-deficient mice are partially but significantly protected in the kidney IRI model and discussed a direct effect of necroptosis in kidney tubules [31]. It is beyond this investigation to identify the detailed mechanism of how RUBCN-deficiency triggers MLKL-mediated necroptosis in this setting, but it is important to realize that pyroptosis does not appear to be involved. It was recently demonstrated that deletion of rubicon from one specific compartment, the renal tubular epithelial cells, did not affect the outcome of kidney ischemia-reperfusion injury [35]. Despite different mouse strains used in that study compared to the one presented here, we interpret these data to be in keeping with our fundings.

As clear as the data on AKI in RUBCN-deficient and *Rubcn/Mlkl*^*DKO*^ mice are, several open questions remain unanswered. In contrast to previous models, we did not detect signs for the development of autoimmunity in *Rubcn*^*KO*^ mice [29]. While we do see a failure to develop normal body weight in the RUBCN-deficient and the combined RUBCN/GSDMD-deficient male and female mice, we do not see elevated levels of antinuclear antibodies (ANAs) or autoantibodies against double-stranded DNA. Importantly, we detected lower spleen weight in *Rubcn*^*KO*^ female mice compared to control female mice, while no differences were observed in the males. Why RUBCN-deficient mice fail to gain normal body weight in comparison to littermates, therefore, remains an open question.

Another limitation of our study is the lack of explanation of the progressing chronic kidney disease in these mice. In particular, we observed higher urea levels in the RUBCN-deficient male and female mice, alongside an increase in urine protein-to-creatinine-ratio (proteinuria) exclusively in the male mice. We did not observe any typical signs of glomerulonephritis.

In conclusion, we present evidence for *Rubcn*^*KO*^ mice to be significantly sensitive to kidney IRI, an effect that is partially reversed on a combined MLKL-deficient background. In addition, we identify the RUBCN-deficient mice as mildly affected by chronic kidney disease over the first year of life, but we did not detect the development of a severe autoimmune phenotype.

## Supplementary information

Figure S1

Figure S2

Figure S3

Figure S4

Figure S5

Sigure S6

Reproducibility checklist

## Data Availability

All data needed to evaluate the conclusions in the paper are present in the paper and/or the Supplementary Materials.
