# Supplementary figures and images for: Rubicon-deficiency sensitizes mice to mixed lineage kinase domain-like (MLKL)-mediated kidney ischemia-reperfusion injury
Source: Cell Death Dis. 2022 Mar 14;13(3):236. doi: 10.1038/s41419-022-04682-3 (PMC8921192; doi:10.1038/s41419-022-04682-3)

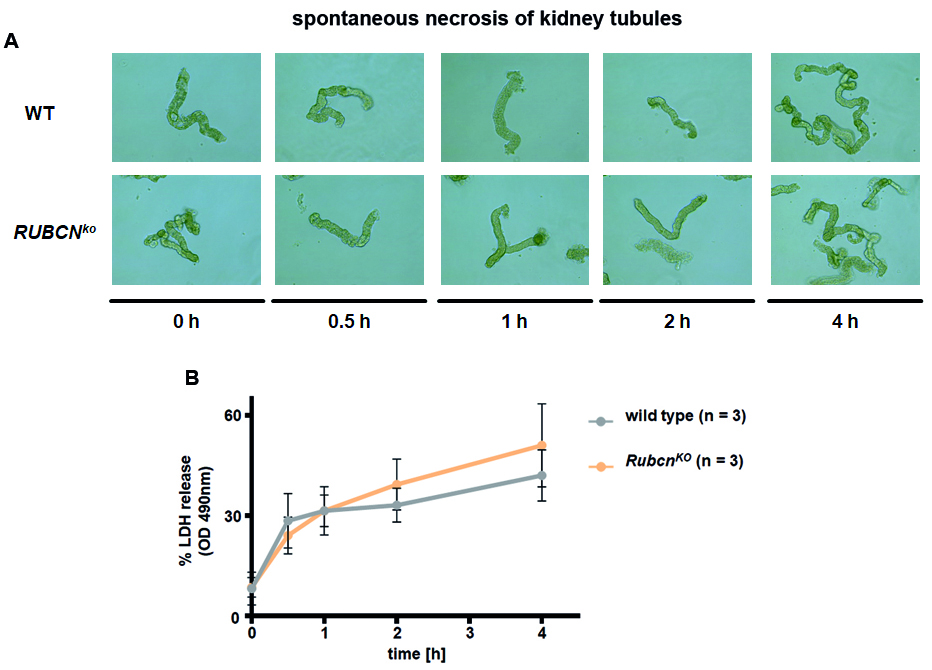

Supplement: Supplementary file 1 — Figure S1 [file 41419_2022_4682_MOESM1_ESM.jpg]

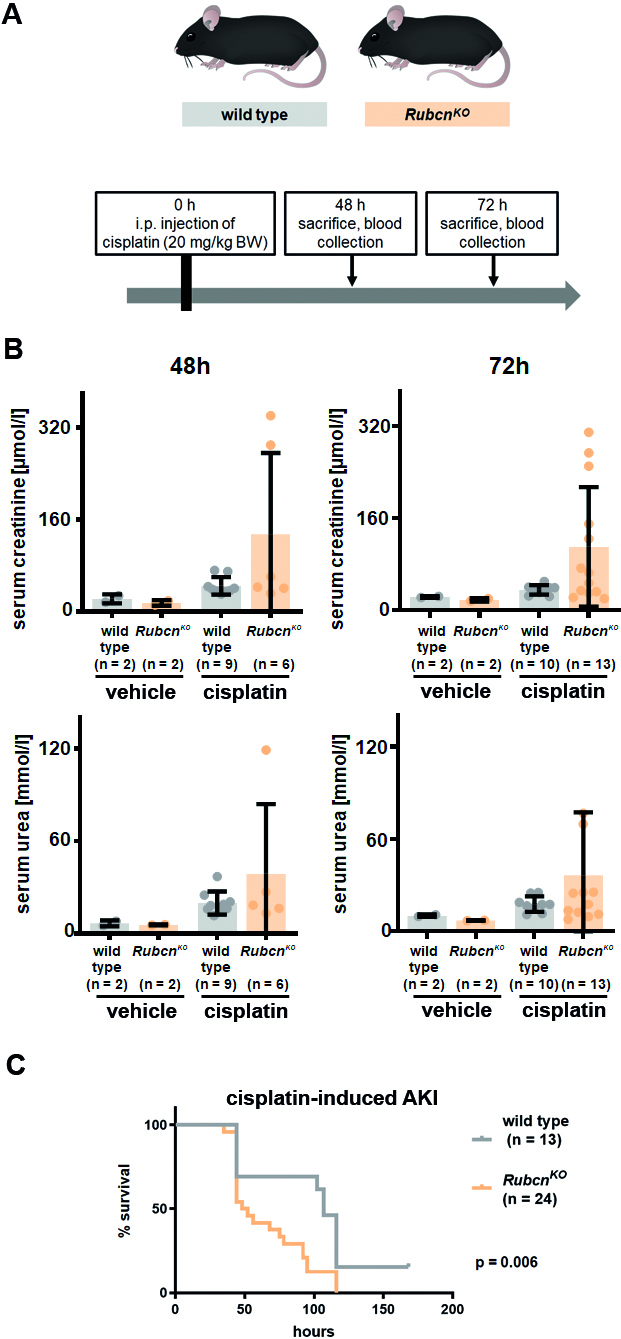

Supplement: Supplementary file 2 — Figure S2 [file 41419_2022_4682_MOESM2_ESM.jpg]

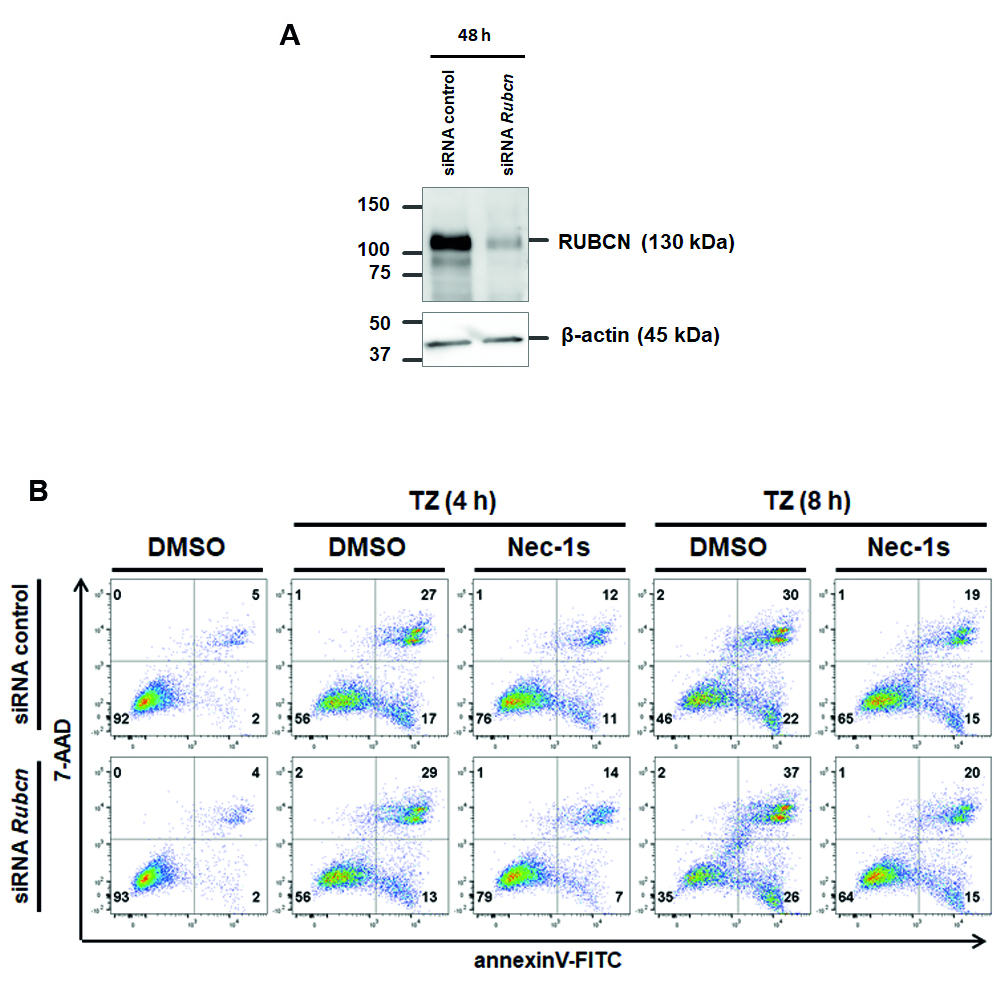

Supplement: Supplementary file 3 — Figure S3 [file 41419_2022_4682_MOESM3_ESM.jpg]

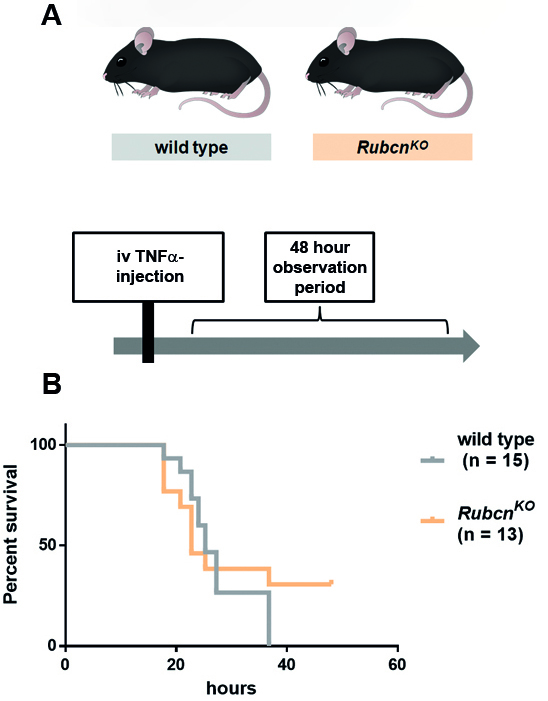

Supplement: Supplementary file 4 — Figure S4 [file 41419_2022_4682_MOESM4_ESM.jpg]

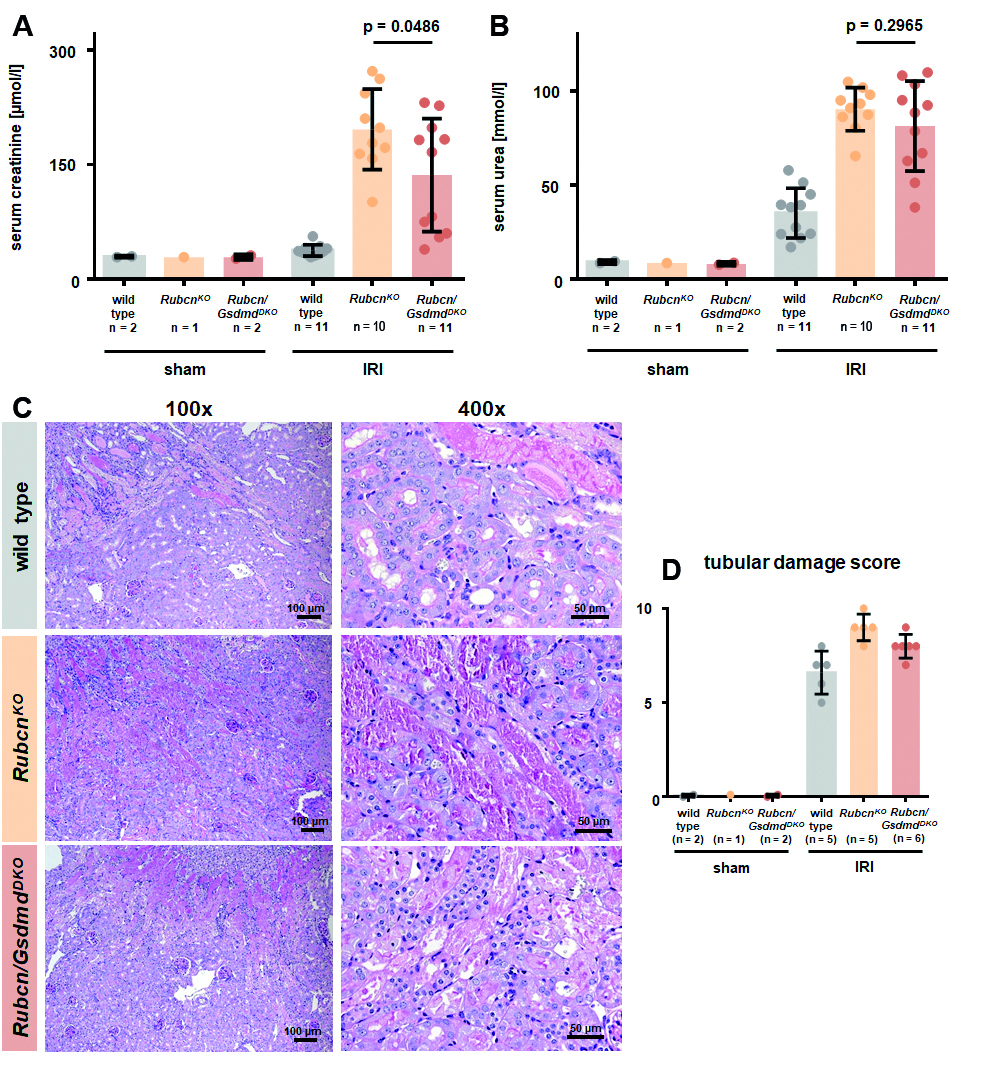

Supplement: Supplementary file 5 — Figure S5 [file 41419_2022_4682_MOESM5_ESM.jpg]

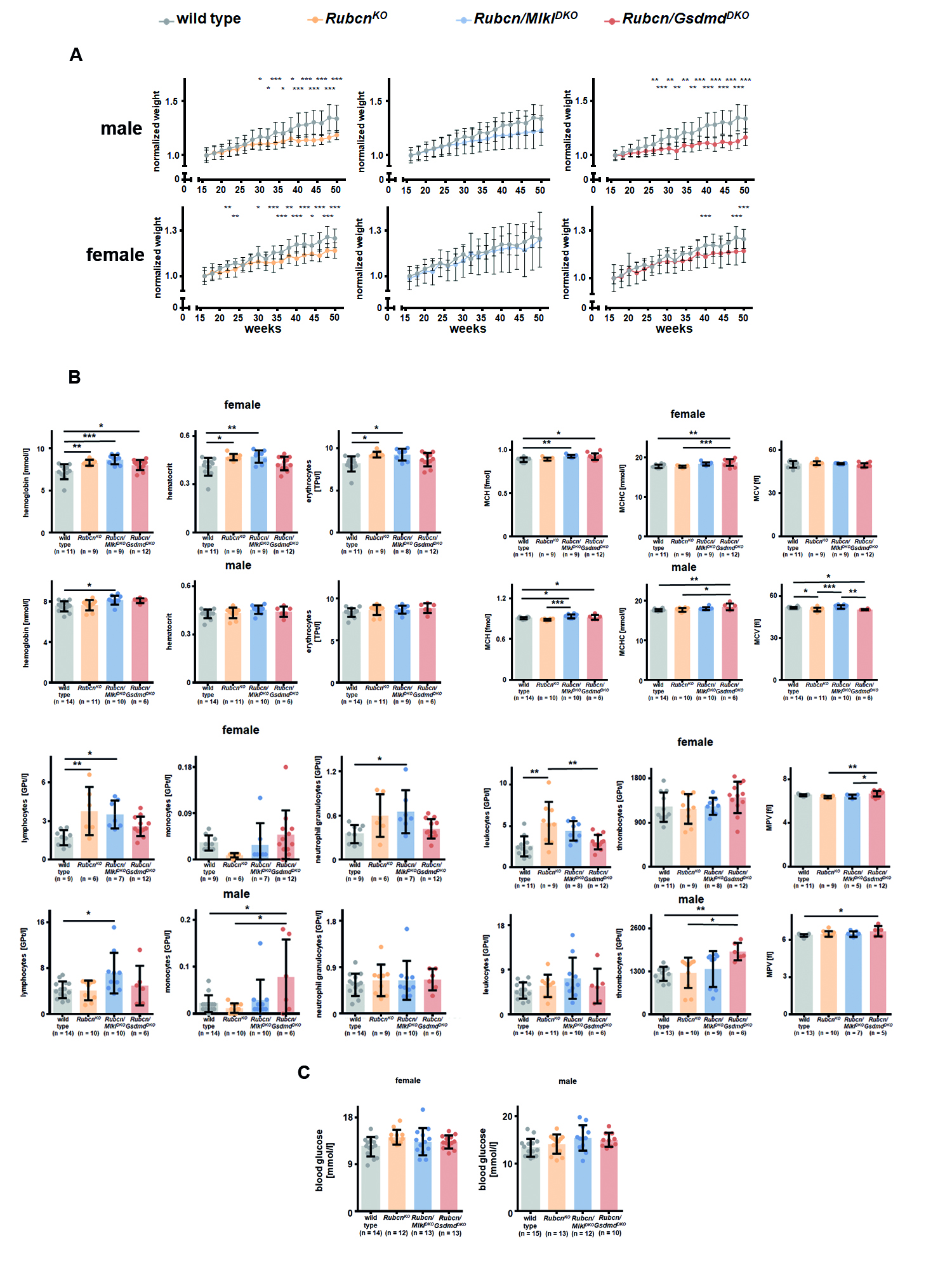

Supplement: Supplementary file 6 — Sigure S6 [file 41419_2022_4682_MOESM6_ESM.jpg]
